# Removal of Micropollutants in Water Reclamation by Membrane Filtration: Impact of Pretreatments and Adsorption

**DOI:** 10.3390/membranes14070146

**Published:** 2024-06-27

**Authors:** Juan C. Aldana, Cristina Agudelo, Pedro M. Álvarez, Juan L. Acero

**Affiliations:** Departamento de Ingeniería Química y Química Física, Instituto Universitario de Investigación del Agua, Cambio Climático y Sostenibilidad (IACYS), Universidad de Extremadura, Avenida de Elvas s/n, 06006 Badajoz, Spain; aldana@unex.es (J.C.A.); cristinaar@unex.es (C.A.); pmalvare@unex.es (P.M.Á.)

**Keywords:** adsorption, organic micropollutants, membrane filtration, nanofiltration, ultrafiltration, water reclamation

## Abstract

Organic micropollutants (OMPs) present in water and wastewater are in the spotlight because of their potentially harmful effects even at low concentrations and the difficulties of their elimination in urban wastewater treatment plants (UWWTPs). This study explores the impact of some membrane filtration processes on the removal of a group of 11 OMPs with an eye on the effects of two pretreatments (i.e., coagulation and adsorption onto powdered activated carbon (PAC)) and the adsorption of OMPs onto the membranes on the overall removal. For this purpose, ultrafiltration (UF) and nanofiltration (NF) experiments were conducted with selected OMPs spiked in ultrapure water and secondary effluents from UWWTPs. It was observed that the adsorption of OMPs onto the membranes was influenced by the characteristics of the membranes, as well as the presence of effluent organic matter (EfOM). Since adsorption was the dominant mechanism for the rejection of OMPs by UF membranes, a study of the adsorption equilibrium of the micropollutants using UF membrane pieces as the adsorbent was conducted. The adsorption isotherms for the most hydrophobic OMPs fitted the Langmuir model. The efficiency of coagulation and powdered activated carbon (PAC) adsorption coupled with UF were also investigated. Both pretreatments alleviated membrane fouling and improved the rejection of organic and inorganic matter. The PAC pretreatment significantly improved the removal of OMPs in the combined PAC/UF process. The best options for achieving reclaimed water with satisfactory physicochemical quality, nearly devoid of OMPs and microorganisms, and suitable for diverse reuse purposes are either the NF treatment or the combination of PAC/UF.

## 1. Introduction

Water scarcity is a global reality in which climate change, population growth, and economic development are involved. In addition to limited quantity, the low quality of water resources is another problem that negatively affects the supply of drinking water [1]. Several factors such as growing urbanization, increased agricultural activities, the use of fertilizers and pesticides, and soil degradation negatively affect the availability of freshwater resources to supply the population [2]. An alternative to reduce the overexploitation of natural resources is to take advantage of the water treated in urban wastewater treatment plants (UWWTPs) and reuse it for different purposes, such as irrigation [3]. It is important to note that the quality of treated wastewater (reclaimed water) must meet certain standards before it can be reused. The European Union (EU) through Regulation 2020/741 has established the minimum quality standards for water reuse considering different quality levels depending on its potential use, specifically for agriculture.

To produce reclaimed water of suitable quality, some wastewater reclamation processes are currently being implemented in UWWTPs [3]. However, an issue of growing concern in wastewater treatment and reclamation is the presence of several organic micropollutants (OMPs) in the effluent, such as pharmaceuticals, personal care products, and industrial chemicals [4,5]. OMPs represent a potential risk to the environment because of their chemical stability and impact on organisms, which may be toxic, producing cancer and mutagenic or teratogenic effects [6]. Aware of this situation, the European Commission keeps an updated watch list of substances to be monitored in water because of their potential risk. In 2022, through Decision (EU) 2022/1307, the previous list from 2020 was modified by adding new substances such as diflufenican, ofloxacin, or clindamycin, while other OMPs such as sulfamethoxazole, trimethoprim, and a set of azole compounds remained in the list. Similarly, in 2022 the US Environmental Protection Agency (EPA) published the Drinking Water Contaminant Candidate List 5 (CCL 5), which includes 66 chemicals, among others, the azole compounds desvenlafaxine, fluconazole, and tebuconazole. In general, these OMPs are not fully eliminated in UWWTPs, which causes their presence in secondary effluents [5,7,8]. As a result, OMPs included in the European watch list and others are disposed into the environment [9,10,11], representing a health issue and a potential environmental risk [12,13]. To go a step further, UWWTPs should include specific treatment processes aimed at achieving the complete removal of OMPs, thus preventing their unsafe disposal into the environment and/or generating reclaimed water that can be reused for different purposes.

Effective treatment methods for removing OMPs from wastewater include advanced oxidation processes (AOPs) such as UV/H_2_O_2_, ozonation, the Fenton reaction, or photocatalytic oxidation [14,15]. Membrane filtration techniques have also been proven efficient at removing OMPs [8,16], showing several advantages over other treatments, such as high efficacy and selective rejection, no need for chemicals, simplicity of installation, and operation at room temperature. However, membrane fouling is a main limitation, hampering the widespread implementation of membrane technologies, such as ultrafiltration (UF) and nanofiltration (NF), in municipal wastewater reclamation. In these processes, size exclusion, electrostatic interactions, and adsorption have been reported as the dominant mechanisms for the removal of OMPs [8,17,18]. In addition, other mechanisms such as cake layer formation might also be of some relevance in OMP removal, as they promote the adsorption of pollutants on the fouling particles [19]. While NF can achieve a high rejection of OMPs, UF generally achieves low rejection because of the large pore size of the membrane. However, since adsorption is an important mechanism to remove OMPs by UF membranes, the rejection of hydrophobic compounds (log K_ow_ > 3) is favored [20,21]. Membrane technologies can be easily integrated with other treatment techniques to improve the removal of OMPs and mitigate membrane fouling [3,8,22,23]. Thus, previous studies reported the use of some wastewater pretreatments to enhance the performance of UF and NF, including coagulation and activated carbon adsorption [24,25,26,27,28]. While coagulation is effective at removing suspended solids, colloids, and high molecular weight (MW) organic compounds, activated carbon adsorption can retain organics within a wide MW range [3].

Most studies on wastewater reclamation by combined membrane processes have focused on either the removal of OMPs or membrane fouling. Thus, fouling mechanisms of membrane filtration processes applied to real water reclamation together with OMP rejection and permeate quality evaluation have been seldom studied so far. In addition, scarce information is available concerning the adsorption equilibria of OMPs onto UF membranes. In this work, a comprehensive study was designed to evaluate membrane fouling, OMP removal, and permeate quality in UF and NF processes. The main objectives were (i) to investigate the impact of UF and NF processes on the removal of a group of 11 OMPs included in the EU watch list; (ii) to assess the effects of two pretreatments (coagulation and adsorption onto PAC) on the adsorption of OMPs onto the membranes; (iii) to analyze the impact of EfOM on the process performance; (iv) to establish the best strategy to remove OMPs from polluted urban wastewater producing a final effluent that can be reused for several purposes.

## 2. Materials and Methods

### 2.1. Materials

GE Sepa™ UF and NF commercial polymeric membranes, denoted by PT and HL, respectively, were supplied as sheets (30.5 × 30.5 cm^2^) by GE Osmonics Labstore (Minnesota, MN, USA). Table 1 summarizes the main properties of these membranes.

Most of the selected OMPs (amoxicillin (AMX), trimethoprim (TMP), desvenlafaxine (DVF), ciprofloxacin (CFX), fluconazole (FLZ), sulfamethoxazole (SMX), imazalil (IMZ), prochloraz (PCZ), tebuconazole (TBZ), penconazole (PNZ), and dimoxystrobin (DTB)) were purchased from Sigma-Aldrich (Burlington, MA, USA). In contrast, fluconazole (FLZ) was obtained from Acros Organics (Geel, Belgium). Micropollutant purity was above 98 wt.% in all cases. Table 2 summarizes some physicochemical properties of the selected OMPs. All these properties were found in PubChem (Bethesda, MD, USA), except log K_OW_, log D, and molar volume, which were obtained from the ChemSpider database from the Royal Society of Chemistry (London, UK). Feed solutions were prepared by spiking the OMPs cited above (1 µM of each compound) into buffered ultrapure (UP) water (2 mM phosphate buffer; ionic strength around 3.5 mM at pH 7 and 6.0 mM at pH 8) or secondary effluents (SE1 and SE2) collected from the outlet of two UWWTPs in South-West Spain. The treatment scheme applied in these UWWTs consisted of pretreatment, biological process, and secondary clarification. After collection, SE1 and SE2 samples were stored in a refrigerator at 4 °C until use. Table 3 summarizes the main characteristics of SE1 and SE2.

The reagents used to prepare phosphate buffer solutions (H_3_PO_4_ and Na_2_HPO_4_) were purchased from Panreac Química (Castellar del Vallès, Spain). HPLC-grade acetonitrile was acquired from Honeywell Riedel-de Häen (Seelze, Germany) while UP water was produced using a Millipore Milli-Q system (Burlington, MA, USA). Ferric chloride, used in coagulation pretreatment, was supplied by Panreac (Castellar del Vallès, Spain), as well as the PAC used for adsorption pretreatment. PAC particles were soaked overnight in UP water and then dried at 105 °C for 2 h. Analysis of this PAC provided a mean particle size of 80 µm, BET surface area of 710.4 m^2^·g^−1^, mesopore surface area of 29 m^2^·g^−1^, micropore surface area of 684.9 m^2^·g^−1^, and average pore diameter of 1.8 nm, which confirmed a predominantly microporous distribution of pore sizes [31].

### 2.2. Filtration Experiments

**Figure 1:**
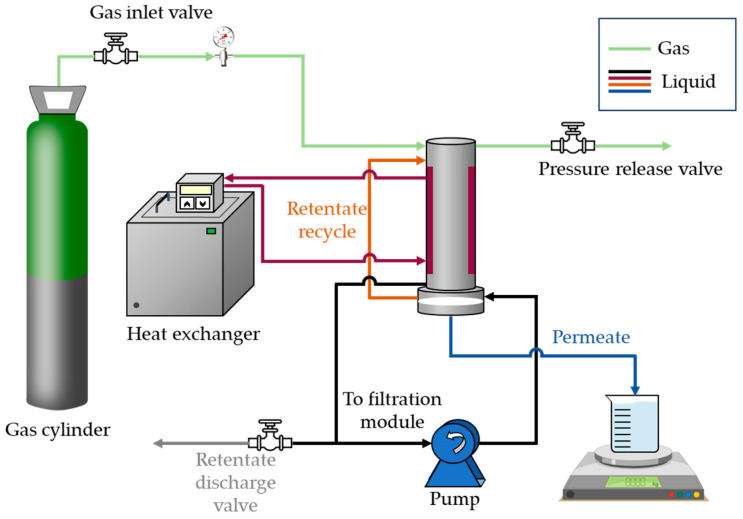
Filtration system CM-CELFA model P-28.

Membrane filtration experiments were conducted using UF PT and NF HL membranes in P-28™ laboratory scale membrane filtration equipment from CM CELFA Membrantrenntechnik AG (Seewen, Switzerland) (Figure 1), which is described elsewhere [32]. Briefly, it comprised a Scherzinger 3000 pump (Gütenbach, Alemania) that fed the water from a 500 mL storage tank to the membrane module tangentially across the surface of the membrane. The operating mode was batch concentration, so that the retentate was recycled to the storage tank and the permeate was withdrawn from the unit. The operating temperature (20 °C) was controlled by means of a digital controller (VWR, model 1166D, Radnor Township, PA, USA). The transmembrane pressure (*TP*) was set at a desired value by pressurizing the storage tank with nitrogen gas. The effective membrane surface was 28 cm^2^, the cross-sectional area 14.85 mm^2^, the feed flow rate 29.84 mL·s^−1^, and the tangential velocity (v) 2 m·s^−1^.

The procedure of a filtration experiment is described elsewhere [33]. Typically, the pristine membrane was soaked in UP water for 24 h and compacted by filtering 200 mL of UP water before the filtration experiment. After setting the desired *TP* according to the membrane type (3 bar for UF and 12 bar for NF), UP water permeate flux (*J_w_*_1_) was determined. Then, the storage tank was filled with 300 mL (*V*_0_) of OMPs spiked in UP/SE, and the sample was recirculated through the system at atmospheric pressure for 2 h to allow for the adsorption of OMPs onto the membrane surface. After this preconditioning stage, the tank was pressurized at the selected *TP*, thus starting the filtration stage. The cumulative permeate volume (*V_P_*) was determined by monitoring the weight of permeate with an analytical balance (Sartorius BL610, Germany), which allowed one to calculate the permeate flux (*J_v_*) across the membrane by Equation (1).

(1) $$J_{V}=\frac{V_{P}}{A\times t}$$

with *A* being the membrane surface area and *t* the filtration time.

Some permeate samples were withdrawn during the experiment to determine the concentration of OMPs and other effluent quality parameters (e.g., COD). The rejection coefficient, *f_i_*, is typically used to estimate the separation achieved by the membrane. It is defined in terms of the values of the objective parameter *P* (e.g., the concentration of a given OMP or COD) in the feed, *P_Ai_*, and in the permeate, *P_Pi_*:

(2) $$f_{i}=\frac{P_{Ai}-P_{Pi}}{P_{Ai}}\times 100$$

The filtration experiment was stopped once a permeate volume of 200 mL was reached, which corresponded to a volume reduction factor of 3 (*VRF*, Equation (3)). Then, the membrane and the system were rinsed three times with UP water for 5 min to eliminate the external fouling from the membrane. Finally, the UP water permeate flux (*J_w_*_2_) was determined again to evaluate the irreversible membrane fouling (internal fouling). All experiments were performed in duplicate.

(3) $$VRF=\frac{V_{0}}{V_{0}-V_{P}}$$

The decrease in the permeate flow in a filtration process can be analyzed using the resistance in series model. In general, the resistance opposing filtration follows Darcy’s Law. Thus, during filtration of UP water, the only resistance opposing the water flow is the resistance inherent to the membrane itself:

(4) $$J_{w}=\frac{TP}{µ\;R_{m}}\;\;\;\;\;\;\;\;\to \;\;\;\;\;\;\;\;R_{m}=\frac{TP}{J_{w}µ}$$

where *J_w_* represents the permeate flow of UP water, *R_m_* corresponds to the hydraulic resistance of the membrane, and µ is the viscosity of water. However, during the filtration of spiked SE or UP water, the permeate flow can be described, in general, by:

(5) $$J_{vss}=\frac{TP}{µ\;R_{t}}\;\;\;\;\;\;\;\;\to \;\;\;\;\;\;\;\;R_{t}=\frac{TP}{J_{vss}µ}$$

where *J_vss_* represents the practically constant permeate flow of SE or UP water with OMPs, *R_t_* corresponds to the total hydraulic resistance, and µ is the viscosity of SE or UP water.

More specifically, *R_t_* is composed of several resistances:

(6) $$R_{t}=R_{m}{+R}_{f}=R_{m}+R_{ef}+R_{if}$$

where *R_f_* is the fouling resistance, which can be considered as the sum of external fouling resistance, *R_ef_*, and internal fouling resistance, *R_if_*. All these resistances can be determined from permeate flow data obtained when filtering UP and SE, using Equations (4)–(6).

### 2.3. Coagulation–Flocculation and Adsorption Pretreatments

Coagulation–flocculation (CF) and adsorption were selected as pretreatments to evaluate their influence on the membrane filtration performance (i.e., permeate flux, membrane fouling, permeate quality, and removal of OMPs). The secondary effluent with higher organic and pathogen loads (SE1) was selected for these experiments.

#### 2.3.1. Coagulation–Flocculation

Experimental conditions used in the CF pretreatment were those proposed in a previous study as optimal [31]. In this case, 130 mg·L^−1^ of ferric chloride was used as a coagulant at the natural pH of SE1. The CF tests were carried out in Velp Scientifica (Usmate Velate, Italy) jar-test equipment, model JLT4, using 600 mL borosilicate glasses. The coagulant dose was added to a beaker containing 500 mL of SE1 spiked with OMP_S_ and was mixed using an agitation speed of 100 rpm for 1 min. Subsequently, the agitation speed was lowered to 30 rpm for 30 min, which allowed the formation of flocs and their gradual growth. Finally, the content of the beaker was left to settle for 60 min, after which the supernatant was recovered and passed through filter paper to eliminate the existing flocs. In this way, 300 mL of pretreated water was separated, characterized, and further used in UF experiments.

#### 2.3.2. Adsorption onto PAC

The dose of PAC used was 50 mg·L^−1^, following recommendations from previous studies [31,34]. Adsorption tests were carried out in a borosilicate jacketed cylindrical vessel (500 mL) thermoregulated at 20 °C using a Fisher Scientific (Loughborough, UK) temperature controller model Isotemp. Five-hundred milliliters of SE1 (fortified with OMPs) was brought into contact with PAC for 24 h at an agitation speed of 120 rpm. At the end of the adsorption process, the obtained suspension was characterized and directly fed to the membrane unit for the subsequent UF step.

### 2.4. Membrane Adsorption Isotherms

Some tests were conducted to evaluate the adsorption equilibrium of OMPs on the hydrophobic UF PT membrane. To do that, test tubes (50 mL) were filled with an aqueous solution containing OMPs (1 μM each, pH 7). Small pieces of the UF PT membrane were added to the tubes and the suspension was stirred in a JP Selecta (Abrera, Spain) Unitronic reciprocal shaking bath at 20 °C. The contact time was at least one week to assure adsorption equilibrium.

The equilibrium isotherm data for the adsorption of the selected OMPs onto the membrane surface were fitted to Langmuir and Freundlich models [35]. Langmuir model can be defined as:

(7) $$q^{*}=\frac{q_{m}K_{L}C^{*}}{1+K_{L}C^{*}}$$

where q^*^ (µg·g^−1^) is the amount of micropollutant adsorbed at equilibrium, *q_m_* (µg·g^−1^) is the maximum monolayer adsorption capacity, *K_L_* (L·µg^−1^) is the Langmuir constant, and C^*^ is the equilibrium aqueous concentration of OMPs.

Similarly, the Freundlich model is defined as:

(8) $$q^{*}=K_{f}{C^{*}}^{1/n\;}$$

where *K_f_* (µg·g^−1^·(L·µg^−1^)^1/n^) is the Freundlich constant and *n* is a constant measuring the reversibility of the adsorption interactions.

### 2.5. Analytical Methods

The concentration of OMPs was analyzed by HPLC using an Agilent 1260 Infinity II apparatus (Agilent, Santa Clara, CA, USA) equipped with a Photodiode Array Detector (DAD). A Phenomenex (Torrance, CA, USA) Kinetex 5 μm C18 100Å 150 × 4.6 mm was used as the stationary phase while the mobile phase was a mixture of aqueous orthophosphoric acid (10 mM) and acetonitrile. A gradient mode analysis was used as shown in Figure S1. The injection volume was 100 µL.

Some characteristics of SEs were determined as shown in Table 3. Electric conductivity (EC) and pH were measured using a Hanna HI255 apparatus (Smithfield, RI, USA). Chemical oxygen demand (COD), total nitrogen (N), and total phosphorous (P) concentrations were determined using Hanna analytical kits (Smithfield, RI, USA). Turbidity was evaluated using a Hanna HI98703 turbidimeter (Smithfield, RI, USA). Dissolved organic carbon (DOC) was analyzed on a TOC-multi N-C 3100 Analytic Jena device (Jena, Germany). All spectrophotometric measurements were performed on a Thermo Scientific Evolution 300 apparatus (Waltham, MA, USA). Microorganism analyses were conducted according to standard procedures (UNE ISO 9308-1 [36] for total coliforms and *E. coli* and UNE ISO 11731 [37] for *Legionella* spp.).

## 3. Results and Discussion

### 3.1. Adsorption of OMPs on a UF Membrane

**Figure 2:**
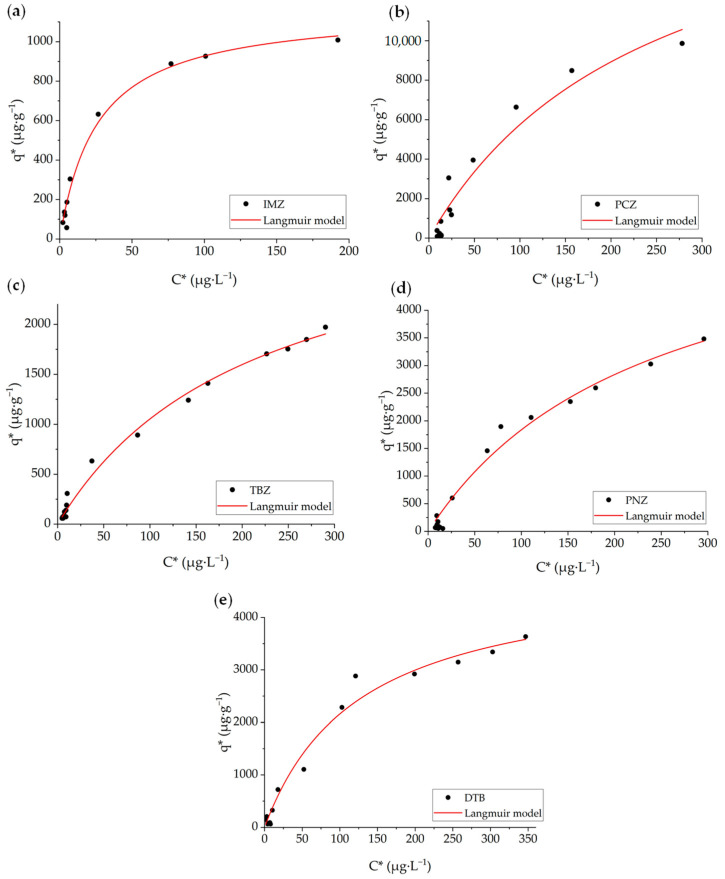
Adsorption isotherms of OMPs on the PT membrane (UF): (**a**) IMZ, (**b**) PCZ, (**c**) TBZ, (**d**) PNZ, and (**e**) DTB. Symbols represent experimental results and lines fitting to the Langmuir model.

To examine the adsorption of OMPs onto the membrane surface, equilibrium isotherms were determined only for the PT membrane because of its higher hydrophobicity and adsorption capacity than the HL membrane (see below). Figure 2 shows the experimental data (symbols) obtained for the most hydrophobic OMPs (IMZ, PCZ, TBZ, PNZ, and DTB). The adsorption of these OMPs exhibits a steadily increasing trend within the concentration range investigated. When fitting data to equilibrium isotherm models via non-linear least squares regression, the Langmuir model fitted reasonably well (R^2^ > 0.93), whereas Freundlich resulted in lower R^2^ values in all cases (Table 4). Therefore, and according to the Langmuir model adsorption mechanism, the micropollutants form a monolayer on the membrane surface [35]. From the values of *q_m_* listed in Table 4, the adsorption capacity order is PCZ > PNZ > DTB > TBZ > IMZ. Since the most hydrophobic compounds (see log K_ow_ values listed in Table 2) were preferably adsorbed onto the membrane surface, hydrophobic interactions between them and the membrane structure seem to be the main adsorption mechanism. Although the adsorption mechanism of hydrophobic partitioning is dominant, the adsorption of organic OMPs with low log K_ow_ values could be accomplished by hydrogen bonding between the membrane polymer and the solute [38]. Hydrogen bonding is a feasible mechanism for CPX adsorption as will be discussed later. The adsorption of the remaining OMPs was rather low, so isotherm data could not be accurately determined.

### 3.2. Coagulation–Flocculation and PAC Adsorption Pretreatments

Considering the potential membrane fouling of EfOM, a series of pretreatments (CF and PAC adsorption) were implemented using SE1 as a water matrix. The resulting effluents after applying these pretreatments showed the characteristics listed in Table 5.

From Table 5, it can be inferred that the CF treatment (SE1-CF experiment) partially removed the organic load of SE1 (around 16–20% removal of COD, DOC, and absorbance at 254 nm), since CF efficiently removes suspended solids, microorganisms, colloids, and hydrophobic and high-MW organic compounds [3]. Furthermore, the removal of total phosphorus was high because of the coagulation of phosphorus compounds forming complexes with Fe^3+^ [31,39]. On the other hand, EC increased slightly because of the addition of ions to the medium. Part of these ions neutralize suspended matter, causing it to precipitate as flocs, while the other part remains in solution, leading to an increase in EC. Likewise, the increase in turbidity is attributed to the presence of micro-flocs that remained as suspended matter after filtration of the supernatant with filter paper. However, there was a significant removal of bacteria, especially of *E. coli*, with only 4 CFU·100 mL^−1^ remaining. Therefore, the effluent generated after this pre-treatment is suitable for reuse in the irrigation of crops that require even reclaimed water quality class A according to Regulation (EU) 2020/741 (e.g., ≤10 CFU·100 mL^−1^ of *E. coli*).

Regarding the adsorption pretreatment with PAC, although the contact time of the secondary effluent with PAC was 24 h, preliminary experiments revealed that 30 min was practically sufficient to reach adsorption equilibrium. The increase in turbidity was attributed to suspended activated carbon, as in this case, the separation of PAC was not conducted before filtration. There was also a significant decrease in organic matter (32% of COD and DOC, and 43% of absorbance at 254 nm), more pronounced than that obtained in the CF pretreatment. PAC achieved great removal efficiency for low-MW pollutants and hydrophobic constituents [3]. Similarly, Gidstedt et al. [40] found higher removals of absorbance at 254 nm than DOC when treating different wastewater process streams by PAC. This is a result of the adsorption of several types of aromatic and hydrophobic organic compounds on the surface of PAC, despite its low dosage [31,41]. However, the removal of microorganisms was rather limited, so the adsorption pretreatment on PAC is not an effective disinfection method, resulting in reclaimed water that cannot be reused for various purposes.

**Figure 3:**
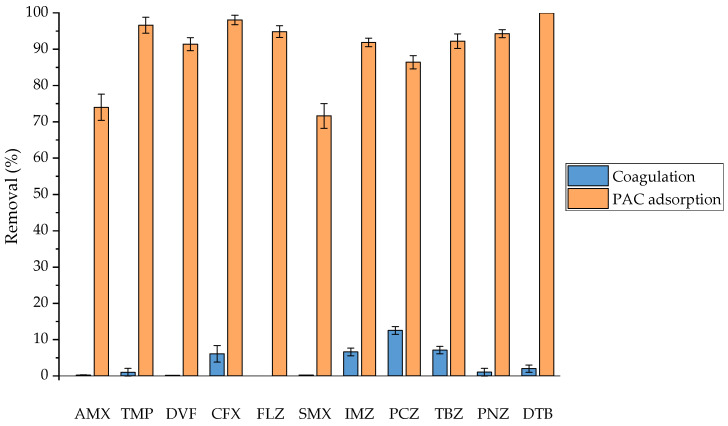
Removal of OMPs from SE1 by CF and PAC adsorption pretreatments.

As is apparent from Figure 3, the selected OMPs were relatively easily removed by the PAC adsorption pretreatment, with their concentration reduced by approximately 75–95% in all cases. Compounds with high log K_ow_ values were preferably adsorbed, and therefore the adsorption of OMPs on PAC can mostly be explained by hydrophobic partitioning [34]. In addition, electrostatic interactions were also important for hydrophilic OMPs, since non-charged compounds at pH 8 (CFX, TMP, and FLZ) were adsorbed to a higher degree than negatively charged compounds (AMX and SMX). Therefore, the negatively charged hydrophilic OMPs were generally less adsorbed and suffered a greater impact from EfOM [23,34,42]. On the other hand, the CF pretreatment led to low (CFX, IMZ, PCZ, TBZ, PNZ, and DTB) or negligible (AMX, TMP, DVF, FLZ, and SMX) removals. The mechanism of the removal of target compounds by CF is mostly coprecipitation (especially of anionic species) and adsorption [34]. Hydrophobic OMPs (IMZ, PCZ, TBZ, PNZ, and DTB with log K_ow_ > 2.5) may be adsorbed on the surface of iron flocs through hydrophobic interactions. Since the removal of anionic OMPs (AMX and SFX) was negligible, coprecipitation was deduced not to be a dominant mechanism for OMP removal by CF. CFX, which is a hydrophilic compound (log K_ow_ < 1) with a carboxylic acid moiety that is dissociated at pH above 6 (although CFX is in the form of a zwitterion at circumneutral pH), deserves special attention. The negative carboxylic ion of CFX may be electrically aggregated with Fe^3+^ ions in the CF process [43] or may interact with the EfOM present in SE1. Since the EfOM can be removed by CF, another mechanism for OMP removal to consider is their association with EfOM via complexing or hydrogen bonding [22].

### 3.3. Filtration Experiments

A series of UF and NF experiments were conducted with OMPs spiked in UP water at two different pH levels (pH 7 and 8) within the typical pH range of SEs. Permeate flux, membrane fouling, and contaminant removal were analyzed to assess their effectiveness. Later, experiments with UF PT and NF HL membranes were carried out with the selected OMPs spiked in SE1 and SE2. Finally, effluents obtained after CF and PAC adsorption of SE1 were filtered through the UF PT membrane (CF/UF and PAC/UF experiments).

#### 3.3.1. Permeate Flux and Analysis of Resistance in Series

The evolution of permeate flux over filtration time was analyzed throughout the experiments. The results of the normalized permeate flux (*J_v_J_w_*_1_) versus VRF are shown in Figure S2 for PT and HL membranes. In general, there was an initial decrease in permeate flux as the VRF increased. After a certain time (equivalent to a specific value of *VRF*), the flux reached a practically steady value until the end of the experiment. In the case of UP water, the fouling effect was low. The initial drop of permeate flux was likely due to concentration polarization and fast adsorption of OMPs in the initial stages of the process, both on the surface and inside the pores of UF PT and NF HL membranes [44]. The practically constant value of *J_v_* is referred to as the steady-state permeate flux (*J_vss_*), which in this study occurs at *VRF* values around 1.6 and 2.0 for PT and HL membranes, respectively. Table 6 summarizes the values of *J_vss_* determined for each membrane along with the ratio of this parameter to the initial permeate flux with UP water, *J_vss_*/*J_w_*_1_, which is an indication of membrane fouling. It can be observed that fouling was slightly higher in the NF HL membrane since the dominant external fouling mechanism (cake layer formation) is more severe for membranes with smaller pore sizes [45]. Regarding the effect of pH, a slight negative pH influence on the permeate flux can be observed, as the fouling levels reached were slightly higher in the experiments performed at pH 8 for both membranes.

The results obtained for *J_vss_* and the ratio of *J_vss_*/*J_w_*_1_ in UF and NF tests conducted with secondary effluents are also presented in Table 6. Compared to the *J_vss_*/*J_w_*_1_ values obtained with UP water, a slight increase in membrane fouling was observed, likely due to the additional contribution of cake formation or pore-blocking phenomena caused by the presence of organic and inorganic substances in the secondary effluents [22]. Additionally, the decline in permeate flux was somewhat higher for the SE1 effluent, especially with the NF HL membrane, because of its higher degree of contamination.

Results from the UF stage performed after the CF pretreatment (CF/UF experiment) and adsorption using PAC (PAC/UF experiment) on the SE1 effluent are shown in Table 6 as well. It can be observed that in the UF experiments with pretreatment, fouling of the PT membrane was significantly reduced compared to the experiment without pretreatment, reaching *J_vss_*/*J_w_*_1_ values of 0.92–0.94. In the case of CF pretreatment, the removal of colloidal matter likely prevented the formation of a cake layer on the membrane surface, thereby hindering the adsorption of OMPs on it. On the other hand, the presence of PAC, which had previously adsorbed some of the organic matter, facilitated the drag of colloidal particles and promoted continuous membrane cleaning, despite the high concentration of suspended solids present [31].

After performing the filtration experiments, an external cleaning stage of the membrane was carried out as described in Section 2.2. Subsequently, the permeate flux with UP water after the cleaning stage, *J_w_*_2_, was determined, and the values of the *J_w_*_2_/*J_w_*_1_ ratios were calculated, yielding the results shown in Table 6. The closer the value of the *J_w_*_2_/*J_w_*_1_ ratio is to unity, the lower the internal (irreversible) fouling, primarily due to pore blocking and adsorption of micropollutants [46]. On the other hand, a greater difference between *J_vss_*/*J_w_*_1_ and *J_w_*_2_/*J_w_*_1_ ratios implies the existence of greater external fouling (reversible). Based on the *J_w_*_2_/*J_w_*_1_ values, it can be inferred that, for NF membranes, fouling was primarily external and was almost completely removed with the surface wash. However, for UF PT membranes, fouling was not eliminated with washing and caused an irreversible decrease in permeate flux. The adsorption of hydrophobic solutes onto the UF PT membrane promoted internal and irreversible fouling due to adsorption and pore blockage and hindered performance [47].

In the UF experiments conducted after CF or adsorption on PAC steps, the permeate flux was recovered almost entirely after a superficial membrane wash. Therefore, the application of these pretreatments significantly alleviated the fouling of UF membranes, especially internal fouling, thus reducing the frequency of cleaning and contributing to an increase in their lifespan [26].

Table 7 presents the results obtained from the analysis of resistances in series for the experiments conducted with UP water and secondary effluents. The parameter *R_f_*/*R_t_*·100 has also been determined, representing the percentage contribution of fouling resistance to the total resistance. The main resistance observed was the membrane resistance itself, which is an order of magnitude higher than fouling resistance.

The contribution of *R_if_* and *R_ef_* was rather similar in UF PT membranes. Irreversible fouling due to pore blockage caused by particles in the secondary effluent and the adsorption of micropollutants was important. However, for the NF HL membrane with a smaller pore size, the particles faced greater difficulty penetrating, making external fouling predominant. Thus, in NF membranes, concentration polarization and possible cake formation contributed more to resistance than pore blocking and adsorption. Therefore, internal fouling can be considered practically negligible, implying a non-significant obstruction of the pores of the hydrophilic HL membrane.

The application of CF and PAC pretreatments substantially improved the recovery of UF PT membranes, reducing both internal and external fouling. These results indicate that CF and PAC pretreatments were effective for flux improvement and fouling alleviation. A notable reduction of *R_if_* was observed, which can be explained by the removal of dissolved and colloidal substances entering the membrane pores, thereby reducing the pore blockage [26]. Similarly, an important reduction of irreversible fouling due to the removal of medium- and low-MW fractions was also observed by Cheng et al. [26] in the treatment of natural water by PAC/UF.

#### 3.3.2. Rejection of Micropollutants

**Figure 4:**
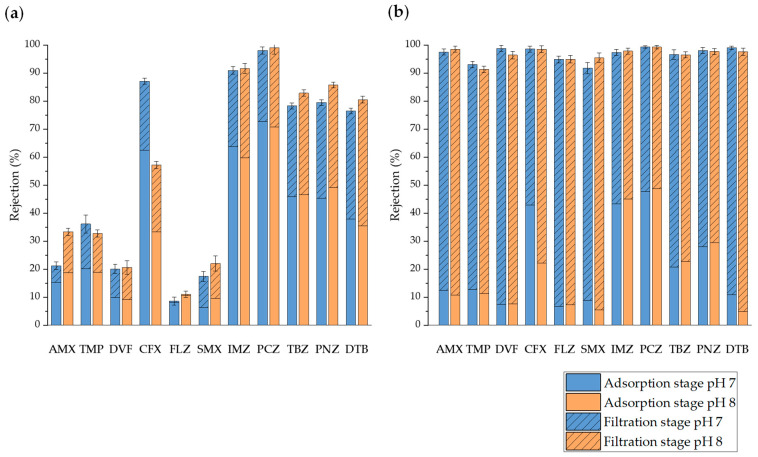
Rejection of OMPs in UP water with the UF PT membrane (**a**) and the NF HL membrane (**b**).

The effectiveness of the filtration process was quantified using the rejection coefficients (*f_i_*) for each micropollutant (see Equation (2)). Figure 4 displays the values of the rejection coefficients for OMPs in the experiments conducted with UP water by using UF PT and NF HL membranes. Adsorption onto the membrane before filtration can be distinguished from the total rejection after filtration (*VRF* = 3). As observed in the figures, the rejection coefficients cover a wide range of values, reflecting the influence of various membrane characteristics (MWCO and hydrophobicity) and solute properties (molecular volume, pK_a_, log K_ow_, dipole moment, etc.) that affect rejection through different mechanisms (adsorption, steric effects, electrostatic repulsion, etc.).

It can be observed that the pre-adsorption of OMPs was higher in UF experiments with the hydrophobic PT membrane than in NF experiments with the hydrophilic HL membrane [48]. Additionally, the pre-adsorption on both membranes was higher for more hydrophobic OMPs according to the log K_ow_ and log D at pH 7.4 values (Table 2): DTB, PCZ, PNZ, IMZ, and TBZ. Although the experimental conditions applied in the experiments performed to determine adsorption isotherms and filtration experiments were different, there was a reasonable correspondence between the results obtained for the favorable adsorption of the most hydrophobic compounds. Thus, the adsorption isotherms described above showed that PCZ had the highest surface interaction with the UF PT membrane. On the other hand, the adsorption of CFX was also significant despite not being a hydrophobic compound (log K_ow_ < 1), indicating the effect of other types of interactions with the membrane surface (e.g., hydrogen bonding between CPX and the membrane surface), especially at pH 7. CFX is more soluble when it is present as an ion [47]. At neutral pH, CFX is in its zwitterion form, making it less soluble in water. As the pH increases, the anionic form of CFX^−^ predominates, thus increasing CFX solubility. This explains the significant decrease in the adsorption and rejection at pH 8 compared to pH 7 regardless of the type of membrane used. In general, little adsorption was observed for OMPs with low log K_ow_ values, with the adsorption of negatively charged OMPs being even lower than that of neutral ones, which can be attributed to the repulsion of negatively charged compounds by the membrane, as previously reported by Dagher et al. [18] in a study performed with 164 pesticides and their metabolites.

The final rejection with the PT membrane was high for the previously mentioned more hydrophobic OMPs. The remaining micropollutants (AMX, TMP, DVF, FLZ, and SMX) exhibited relatively low rejection values with the PT membrane due to their hydrophilic nature (Table 2). Therefore, the main rejection mechanism for OMPs was their adsorption onto the hydrophobic UF PT membrane. In UF membranes, the contribution of the size exclusion or steric hindrance mechanism to the rejection of these OMPs must be less important, as the MW of the micropollutants was lower than the MWCO of the membrane. In any case, these mechanisms may occur mainly after membrane saturation with OMPs [44]. However, the final rejection of all OMPs was close to 100% with the NF HL membrane. According to these results, the primary rejection mechanism for micropollutants with the NF HL membrane must be size exclusion, especially for the OMPs with weaker polarity and a higher MW or molar volume, whose adsorption and diffusion in the membrane were less important [18,49]. Thus, the compound with the lowest rejection at pH 7 was SMX because its MW and molar volume were smaller than the others (Table 2).

Regarding the influence of pH, the rejection of AMX and SMX slightly increased at pH 8, a condition at which both micropollutants were dissociated and negatively charged, causing electrostatic repulsion with the negatively charged UF PT and NF HL membrane surface [18,34,45]. However, the rejection of TMP (pK_a_ = 7.12) was slightly higher at pH 7 since its positive charge at pH < pK_a_ enhanced the contribution of electrostatic attractions with the membrane surface [45]. There were minor variations in the rejection percentage of the rest of the OMPs with pH, except for CFX, which exhibited higher rejection at pH 7 than at pH 8 with the PT membrane. Consequently, there is a small contribution of electrostatic interactions between neutral OMPs and the membrane surface, with their rejection likely being caused by hydrophobic interactions and size exclusion.

**Figure 5:**
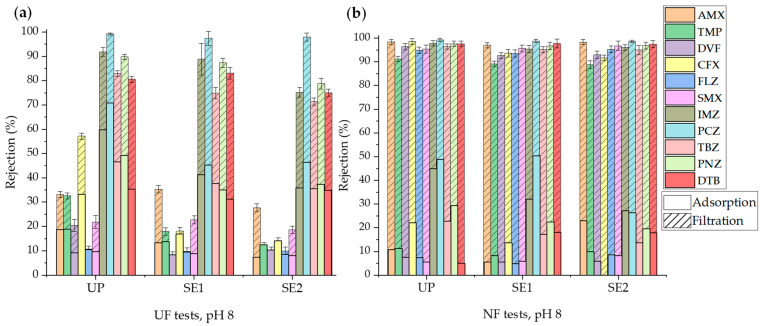
Rejection of OMPs in UF (**a**) and NF (**b**) experiments with different water matrices.

The rejection of OMPs in UF and NF experiments of secondary effluents is depicted in Figure 5. Similar graphs for individual OMPs are included in Figure S3. The results obtained in an experiment with UP water at pH 8 (close to the pH of the secondary effluents) are also included for the sake of comparison. There was a higher removal of OMPs in UP water than in SEs with the UF PT membrane because the EfOM present in SEs competed with micropollutants to be adsorbed on the membrane surface, with adsorption being the primary mechanism for retaining OMPs in the UF PT membrane. Additionally, a slightly higher removal of OMPs was observed in SE1 than in SE2, probably because of hydrophobic or electrostatic interactions between the EfOM, more present in SE1, and the micropollutants [45,50]. In addition, matrix constituents could form a cake layer on the membrane surface that interacts with OMPs, improving their rejection [22]. However, the rejection of OMPs by the NF HL membrane was similar in all three experiments. Therefore, the additional presence of organic and inorganic matter in SEs did not significantly affect the selectivity of the NF HL membrane. These results corroborate that size exclusion is the primary mechanism for retaining micropollutants in the NF process.

**Figure 6:**
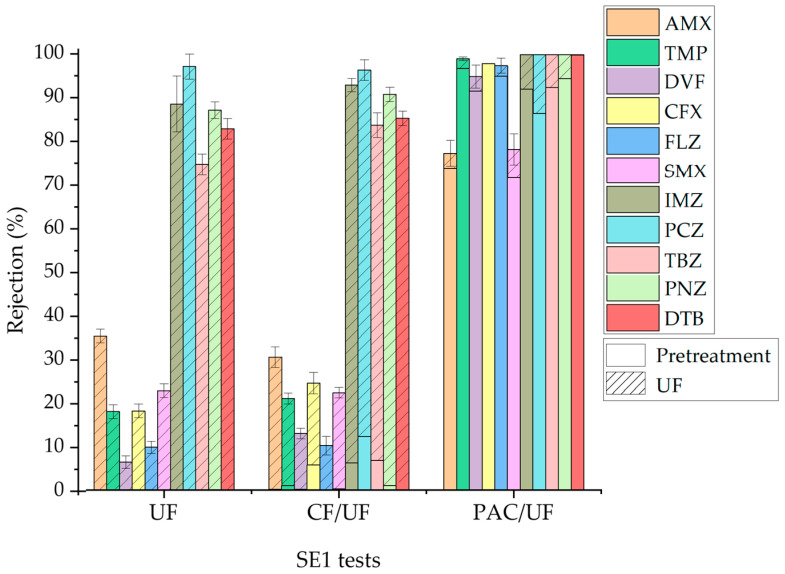
Rejection of OMPs in the UF of SE1 and total removal in the combined treatments CF/UF and PAC/UF.

Figure 6 illustrates the total removal of OMPs achieved in combined treatments, consisting of a CF or adsorption on PAC pretreatment followed by a UF step with the PT membrane (CF/UF and PAC/UF, respectively) applied to the SE1 effluent. The figure also includes the rejection of OMPs in a single filtration experiment for the sake of comparison. Similar graphs for individual OMPs are included in Figure S4. It can be observed that the CF stage did not significantly influence the overall removal of OMPs because of their low rejection in the pretreatment. On the other hand, the adsorption pretreatment on PAC significantly improved the removal of OMPs in the combined process compared to UF alone, achieving near full removal of all OMPs except AMX and SMX, which exhibited removal percentages close to 80%. The removal of OMPs by the PAC/UF process was also higher than that obtained by single PAC adsorption. The combination of PAC with tight UF membranes has been proven to be a better option for OMP removal than the treatment scheme based on UF and reverse osmosis [23].

#### 3.3.3. Quality of the Reclaimed Water

**Figure 7:**
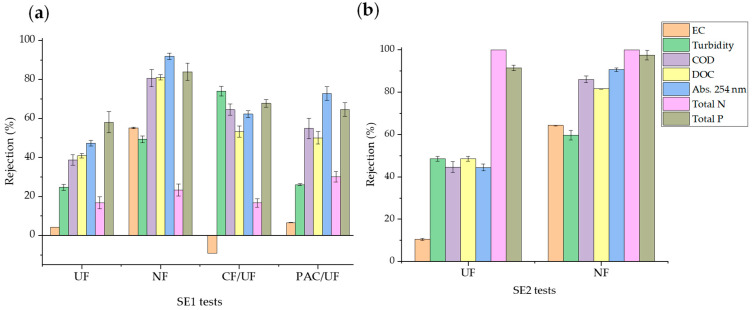
Rejection of physicochemical parameters in the filtration of SE1 (**a**) and SE2 (**b**).

Figure 7 displays the percentage removal of some physicochemical parameters as obtained in UF and NF experiments conducted with SE1 (a) and SE2 (b), respectively. It can be observed that EC was retained much more in the NF HL membrane than in the UF PT membrane for both SEs, as NF membranes can retain multivalent salts such as carbonates, sulfates, phosphates, etc. The rejection of turbidity was also higher with the NF membrane. In this regard, it is worth noting that in filtration with UF PT and NF HL membranes, the entirety of suspended particles was generally retained. Organic matter, expressed as COD, DOC, and absorbance at 254 nm, was retained at around 40–45% with the UF PT membrane for both SEs. In contrast, higher rejections exceeding 80% were achieved with the NF HL membrane because of its smaller pore size, preventing the passage of molecules of a certain size (>150 Da). Finally, since the SE2 effluent had very low N and P content, similar rejections were obtained with both membranes. However, the rejection of N and P was higher with the NF HL membrane in the case of the SE1 effluent. The values reported by Hube et al. [16] for the rejection of physicochemical parameters in the direct membrane filtration of different municipal wastewater samples were similar to those obtained in the present study.

Additionally, it can be observed that when the CF pretreatment was applied (Figure 7a), the total removal of organic matter was greater than that obtained by single UF, increasing the removal of COD, DOC, and absorbance at 254 nm from 40% to around 60%. The CF stage also improved the effluent quality in terms of turbidity and phosphorus reduction. These results can be explained by the removal of suspended solids and macromolecules (mainly hydrophobic compounds of higher MW), as well as phosphates that easily interact with the coagulant [8,16]. However, the EC of the resulting permeate was higher than that of SE1 because of the residual presence of ions from the coagulant. On the other hand, the adsorption pretreatment with PAC slightly increased the removal of organic matter, with a more noticeable increment of the removal of absorbance at 254 nm due to the adsorption of low molecular weight aromatic compounds on the PAC [3]. Likewise, a slight increase in the removal of nitrogen and phosphorus was observed in the combined PAC/UF process.

Finally, Table 8 details some characteristics of the permeates collected in the different single and combined treatments applied to secondary effluents SE1 and SE2. Overall, the quality of the final permeates improved when the UF stage was applied after a CF or adsorption with PAC pretreatment. In addition, the quality of effluents obtained by CF/UF or PAC/UF was better than that obtained by a single CF or PAC treatment (results in Table 5) because of the removal of EfOM in the UF step. Nevertheless, the permeate generated in the NF treatment had higher quality, mainly because of the low presence of organic matter and EC.

Microbiological analyses of total coliforms, *E. coli*, and *Legionella* spp. revealed that no microorganisms at all were present in the permeates generated in UF and NF experiments. Therefore, the UF PT and NF HL membranes performed disinfection satisfactorily. As reported by Yang et al. [51], even the UF system can efficiently retain microorganisms, including *E. coli*, *Enterococci*, spores of anaerobic sulfite-reducers, and bacteriophages. As a consequence, the resulting permeate has good enough quality to be reused in non-potable applications fulfilling EU Regulation 2020/741.

## 4. Conclusions

The results shown in this work suggest that adsorption plays a significant role in the removal of OMPs by UF membranes. Hydrophobic membranes led to higher adsorption levels, especially for OMPs of a hydrophobic nature. The adsorption isotherms of hydrophobic OMPs fitted well with the Langmuir model. On the other hand, size exclusion was the dominant mechanism for the removal of OMPs by NF membranes. Electrostatic interactions played a minor role in rejecting OMPs by UF and NF membranes. While the contribution of internal (pore blockage caused by the adsorption of OMPs) and external (cake layer) fouling was similar in UF membranes, external fouling caused by concentration polarization and possible cake formation was predominant in NF membranes.

A CF stage slightly improved the performance of a subsequent UF of SEs in terms of increased permeate production, reduced membrane fouling, and enhanced removal of turbidity, total phosphorus, and organic matter. However, it did not show a significant improvement in the removal of the selected OMPs. On the other hand, an adsorption pretreatment with PAC enhanced permeate production and alleviated membrane fouling. Moreover, the PAC pretreatment improved the removal of organic matter, especially aromatic and hydrophobic compounds, and achieved almost complete removal of OMPs. In addition, UF and NF membranes completely retained the analyzed microorganisms (total coliforms, *E. coli*, and *Legionella* spp.).

At the conditions used in this work, the primary choices for SE treatment to produce reclaimed water are either the straightforward NF treatment or the combination of PAC adsorption followed by UF (PAC/UF). Effluents nearly devoid of OMPs and suitable for diverse reuse purposes in compliance with the standards of EU Regulation 2020/741 were obtained. A thorough economic study would help elucidate which of these is the best treatment option considering the cost of PAC and the higher energy requirement of NF. Nonetheless, a third option is the combination of CF followed by UF, which generates a high-quality permeate but incomplete removal of OMPs.

## Figures and Tables

**Table 1:** Properties of the membranes used in this work.

Membrane	Material	MWCO, Da	pH	Contact Angle, ° [20]
PT	PES	5000	2–11	52.8 ± 2
HL	TF	150–300	3–9	30 ± 3

PES: polyethersulfone, TF: thin-film composite.

**Table 2:** Physicochemical properties of selected micropollutants.

OMP	Molecular Formula	Molecular Structure	MW (g·mol^−1^)	pK_a_	log K_ow_	log D (pH = 7.4)	Molar Volume (cm^3^)
Amoxicillin (AMX)	C_16_H_19_N_3_O_5_S	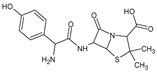	365.40	2.7; 7.2; 9.6 [29]	0.61	−2.72	236.2
Trimethoprim (TMP)	C_14_H_18_N_4_O_3_	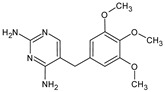	290.32	7.12 [30]	0.79	1.00	231.8
Desvenlafaxine (DVF)	C_16_H_25_NO_2_	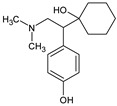	263.38	8.87; 10.1	2.26	0.89	236.1
Ciprofloxacin (CFX)	C_17_H_18_FN_3_O_3_	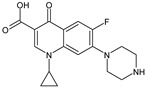	331.34	6.09; 8.62	0.65	−2.23	226.7
Fluconazole (FLZ)	C_13_H_12_F_2_N_6_O	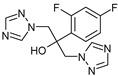	306.27	2.56; 2.94; 11.01	0.50	0.70	205.2
Sulfamethoxazole (SMX)	C_10_H_11_N_3_O_3_S	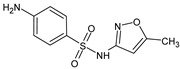	253.28	1.97; 6.16	0.89	−0.56	173.1
Imazalil (IMZ)	C_14_H_14_Cl_2_N_2_O	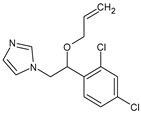	297.20	6.53	3.58	3.94	240.7
Prochloraz (PCZ)	C_15_H_16_Cl_3_N_3_O_2_	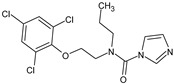	376.70	3.8	3.98	4.08	274.2
Tebuconazole (TBZ)	C_16_H_22_ClN_3_O	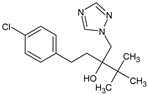	307.82	2.3	3.58	3.74	268.1
Penconazole (PNZ)	C_13_H_15_Cl_2_N_3_	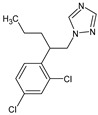	284.18	2.8	3.66	3.88	222.8
Dimoxystrobin (DTB)	C_19_H_22_N_2_O_3_	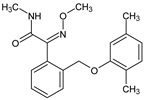	326.40	-	5.08	4.20	297.6

**Table 3:** Physicochemical characteristics of SE1 and SE2.

Parameter	SE1	SE2
pH	8.06	8.19
Electric conductivity (EC) (μS·cm^−1^)	804	831
Turbidity (NTU)	0.73	0.72
COD (mg·L^−1^)	31	15
DOC (mg·L^−1^)	10.49	5.21
Absorbance (254 nm)	0.209	0.117
Total N (mg·L^−1^)	3.0	0.9
Total P (mg·L^−1^)	0.31	0.82
Total coliforms (CFU·100 mL^−1^)	296	210
*E. coli* (CFU·100 mL^−1^)	186	138
*Legionella* spp. (CFU·L^−1^)	ND	ND

ND: not detected.

**Table 4:** Freundlich and Langmuir parameters derived from the adsorption of OMPs on the PT membrane.

Pollutant	Freundlich Model			Langmuir Model		
	K_f_ (µg·g^−1^·(L·µg^−1^)^1/n^)	n	R^2^	q_m_ (µg·g^−1^)	K_L_·10^3^ (L·µg^−1^)	R^2^
IMZ	109.3	2.24	0.910	1168	38.5	0.982
PCZ	134.3	1.27	0.893	19,940	4.06	0.930
TBZ	42.4	1.47	0.991	3311	4.66	0.992
PNZ	53.9	1.35	0.884	6221	4.19	0.979
DTB	128.4	1.72	0.951	4884	7.93	0.981

**Table 5:** Results obtained in the characterization of SE1 before and after each pretreatment.

Expt.	pH	EC (µS·cm^−1^)	Turbidity (NTU)	COD (mg·L^−1^)	DOC (mg·L^−1^)	Absorbance (254 nm)	Total N (mg·L^−1^)	Total P (mg·L^−1^)
SE1	8.06	804	0.73	31	10.49	0.209	3.0	0.31
SE1-CF	7.32	972	1.10	26	8.37	0.169	3.3	0.10
SE1-PAC	8.07	890	2.39	21	7.13	0.120	2.7	0.22
	Total Coliforms (CFU·100 mL^−1^)			*E. coli* (CFU·100 mL^−1^)		*Legionella* spp. (CFU·L^−1^)		
SE1	296			186		ND		
SE1-CF	87			4		ND		
SE1-PAC	223			154		ND		

**Table 6:** Experimental conditions and permeate fluxes obtained in filtration experiments.

Expt.	Membrane	*TP* (bar)	pH	*J_w_*_1_ (L/(h·m^2^))	*J_vss_* (L/(h·m^2^))	*J_vss_*/*J_w_*_1_	*J_w_*_2_/*J_w_*_1_
UP water							
UF1	PT	3	7.00	61.5	54.0	0.88	0.94
UF2	PT	3	8.00	62.1	54.1	0.87	0.90
NF1	HL	12	7.00	90.0	77.3	0.86	0.99
NF2	HL	12	8.00	102.9	87.4	0.85	0.99
SE1							
UF	PT	3	8.06	60.5	51.8	0.86	0.90
NF	HL	12	8.06	90.7	69.4	0.77	0.95
CF/UF	PT	3	7.32	74.6	68.8	0.94	0.97
PAC/UF	PT	3	8.07	72.7	67.1	0.92	0.98
SE2							
UF	PT	3	8.19	63.9	55.1	0.86	0.94
NF	HL	12	8.19	97.2	80.1	0.82	0.99

**Table 7:** Results of the resistances in series analysis.

Expt.	*R_t_*·10^−12^ (m^−1^)	*R_m_*·10^−12^ (m^−1^)	*R_f_*·10^−12^ (m^−1^)	*R_ef_*·10^−12^ (m^−1^)	*R_if_*·10^−12^ (m^−1^)	*R_f_*/*R_t_* (%)
UP water						
UF1	20.1	17.5	2.51	1.34	1.17	12.5
UF2	19.9	17.3	2.57	1.37	1.20	12.9
NF1	55.7	47.9	7.87	6.71	1.16	14.1
NF2	49.3	41.9	7.39	6.50	0.89	15.0
SE1						
UF	20.8	17.8	2.99	1.07	1.91	14.4
NF	62.0	47.5	14.5	11.8	2.73	23.4
CF/UF	15.7	14.4	1.22	0.76	0.46	7.8
PAC/UF	16.0	14.8	1.24	0.89	0.35	7.7
SE2						
UF	19.5	16.9	2.67	1.52	1.15	13.7
NF	53.7	44.3	9.45	8.06	1.38	17.6

**Table 8:** Characterization of permeate samples from UF and NF of secondary effluents.

Parameter	SE1-UF	SE1-NF	SE1-CF/UF	SE1-PAC/UF	SE2-UF	SE2-NF
pH	8.18	7.75	7.34	7.98	8.47	7.52
EC (µS·cm^−1^)	770	361	878	752	744	297
Turbidity (NTU)	0.55	0.37	0.19	0.54	0.37	0.29
COD (mg·L^−1^)	19	6	11	14	8.3	2.1
DOC (mg·L^−1^)	6.20	1.99	4.90	5.26	2.68	0.96
Absorbance (254 nm)	0.110	0.017	0.079	0.057	0.065	0.011
N (mg·L^−1^)	2.5	2.3	2.3	2.1	0.0	0.0
P (mg·L^−1^)	0.13	0.05	0.10	0.11	0.07	0.02

## Data Availability

The raw data supporting the conclusions of this article will be made available by the authors upon request. Some would not be available due to privacy restrictions.

## Supplementary Materials

The following supporting information can be downloaded at: https://www.mdpi.com/article/10.3390/membranes14070146/s1, Figure S1: Chromatographic method information and sample injection of the selected contaminants of emerging concern; Figure S2: Evolution of normalized flux in the experiments performed with (a) UF PT and (b) NF HL membranes; Figure S3. Rejection of (a) AMX, (b) TMP, (c) DVF, (d) CFX, (e) FLZ, (f) SMX, (g) IMZ, (h) PCZ, (i) TBZ, (j) PNZ, (k) DTB in UF and NF experiments with different water matrices; Figure S4. Rejection of (a) AMX, (b) TMP, (c) DVF, (d) CFX, (e) FLZ, (f) SMX, (g) IMZ, (h) PCZ, (i) TBZ, (j) PNZ, (k) DTB in the UF of SE1 and total removal in the combined treatments CF/UF and PAC/UF.
