# Supplementary material for: Removal of Micropollutants in Water Reclamation by Membrane Filtration: Impact of Pretreatments and Adsorption
Source: Membranes (Basel). 2024 Jun 27;14(7):146. doi: 10.3390/membranes14070146 (PMC11278704; doi:10.3390/membranes14070146)
Supplement: Supplementary file 1 [file membranes-14-00146-s001.zip › membranes-3049777-supplementary.pdf]

membranes

Supplementary Materials

Removal of Micropollutants in Water Reclamation by Membrane Filtration: Impact of Pretreatments and Adsorption

Juan C. Aldana, Cristina Agudelo, Pedro M. Álvarez and Juan L. Acero *

Departamento de Ingeniería Química y Química Física, Instituto Universitario de Investigación del Agua, Cambio Climático y Sostenibilidad (IACYS), Universidad de Extremadura, Avenida de Elvas s/n, 06006 Badajoz, Spain
* Correspondence: jlacero@unex.es

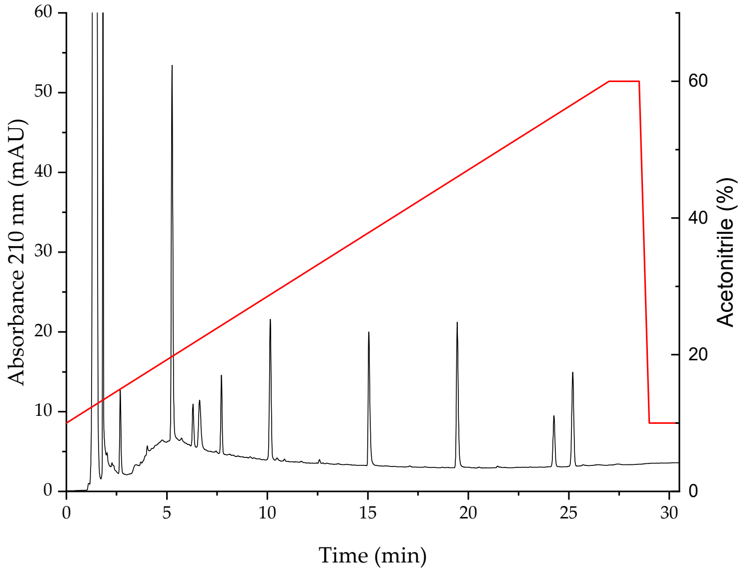

Figure S1. Chromatographic method information and sample injection of the selected contaminants of emerging concern.

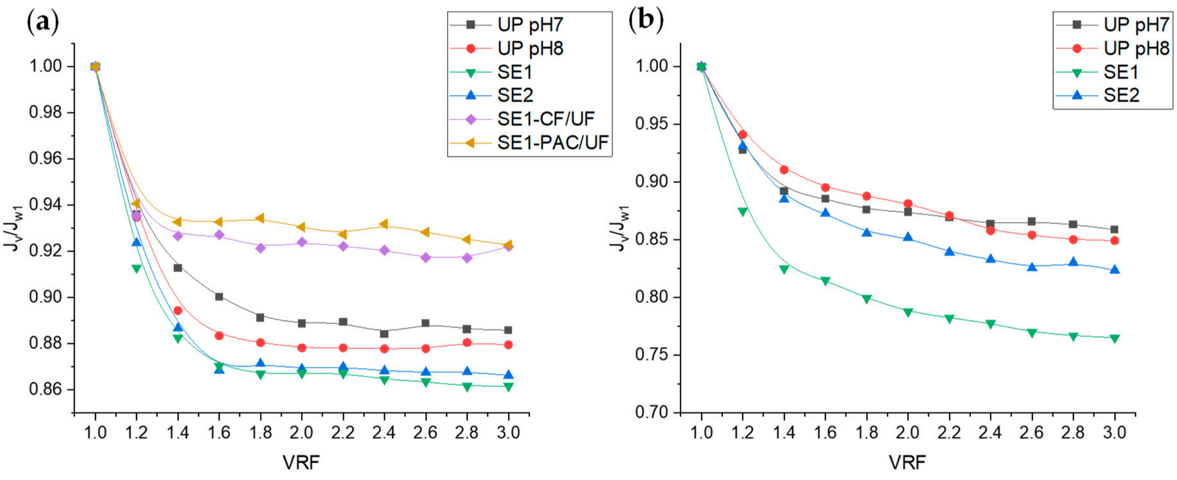

Figure S2. Normalized flux of the experiments using (**a**) ultrafiltration and (**b**) nanofiltration membranes.

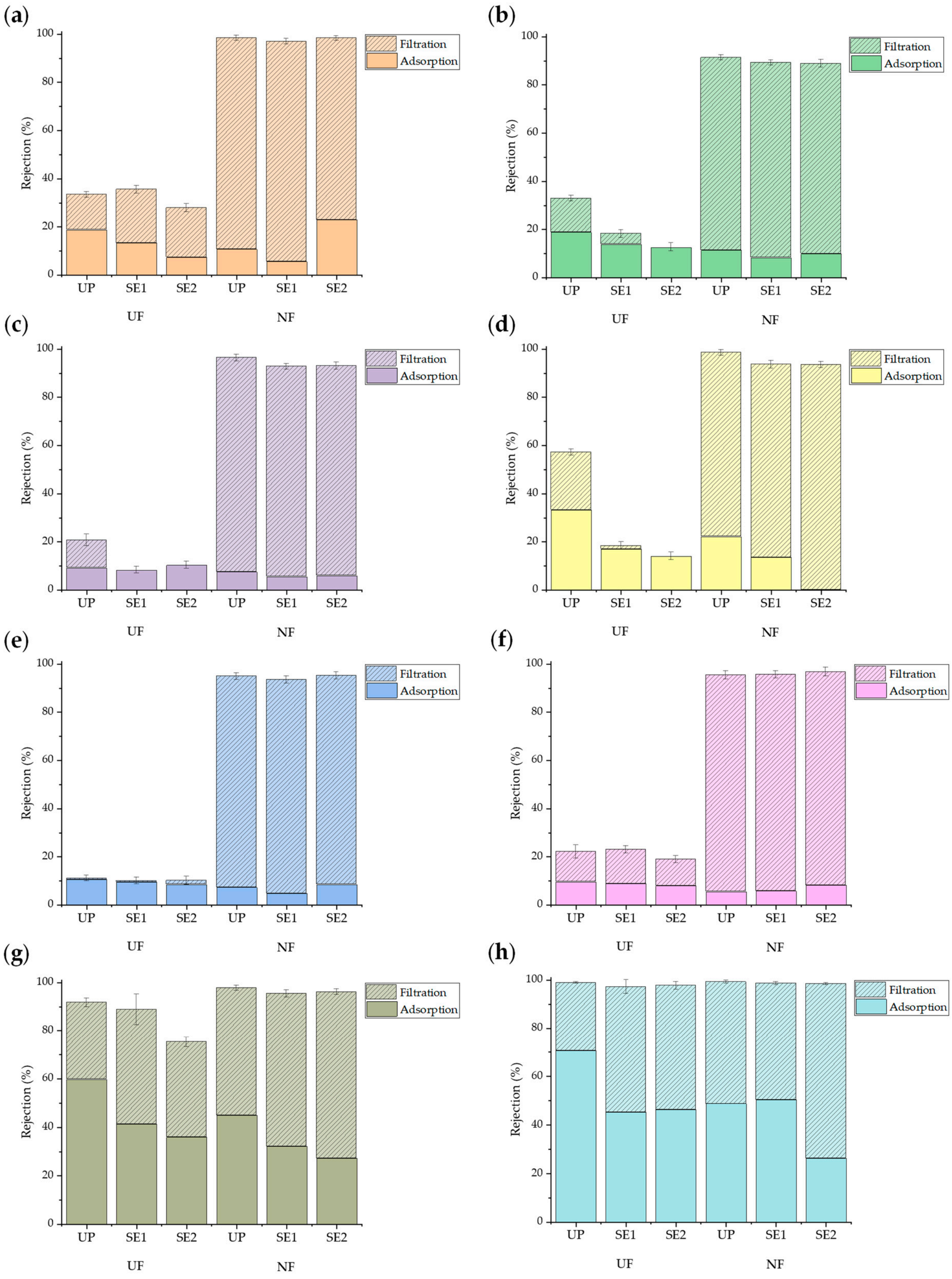

Figure S3. Rejection of (**a**) AMX, (**b**) TMP, (**c**) DVF, (**d**) CFX, (**e**) FLZ, (**f**) SMX, (**g**) IMZ, (**h**) PCZ, (**i**) TBZ, (**j**) PNZ, (**k**) DTB in UF and NF experiments with different water matrices.

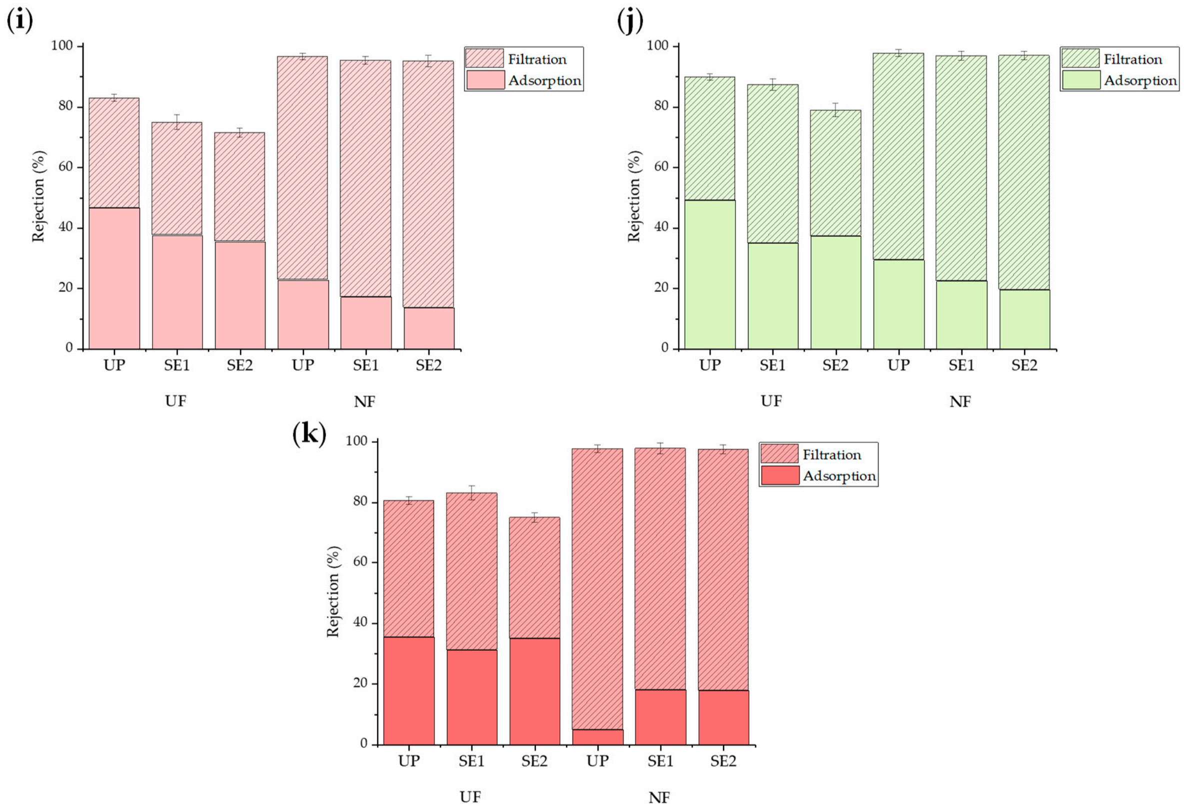

Figure S3 (cont.). Rejection of (**a**) AMX, (**b**) TMP, (**c**) DVF, (**d**) CFX, (**e**) FLZ, (**f**) SMX, (**g**) IMZ, (**h**) PCZ, (**i**) TBZ, (**j**) PNZ, (**k**) DTB in UF and NF experiments with different water matrices.

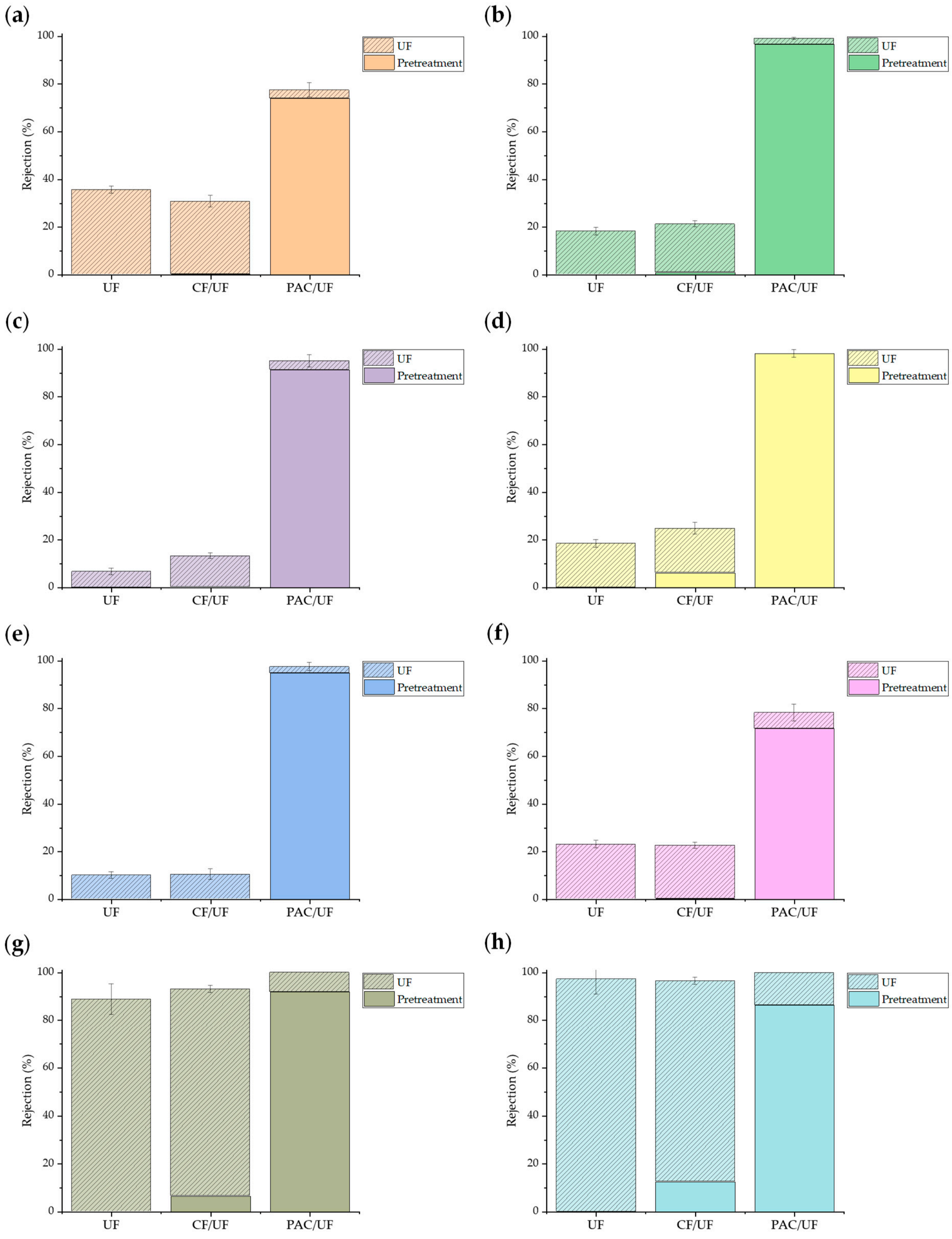

Figure S4. Rejection of (**a**) AMX, (**b**) TMP, (**c**) DVF, (**d**) CFX, (**e**) FLZ, (**f**) SMX, (**g**) IMZ, (**h**) PCZ, (**i**) TBZ, (**j**) PNZ, (**k**) DTB in the UF of SE1 and total removal in the combined treatments CF/UF and PAC/UF.

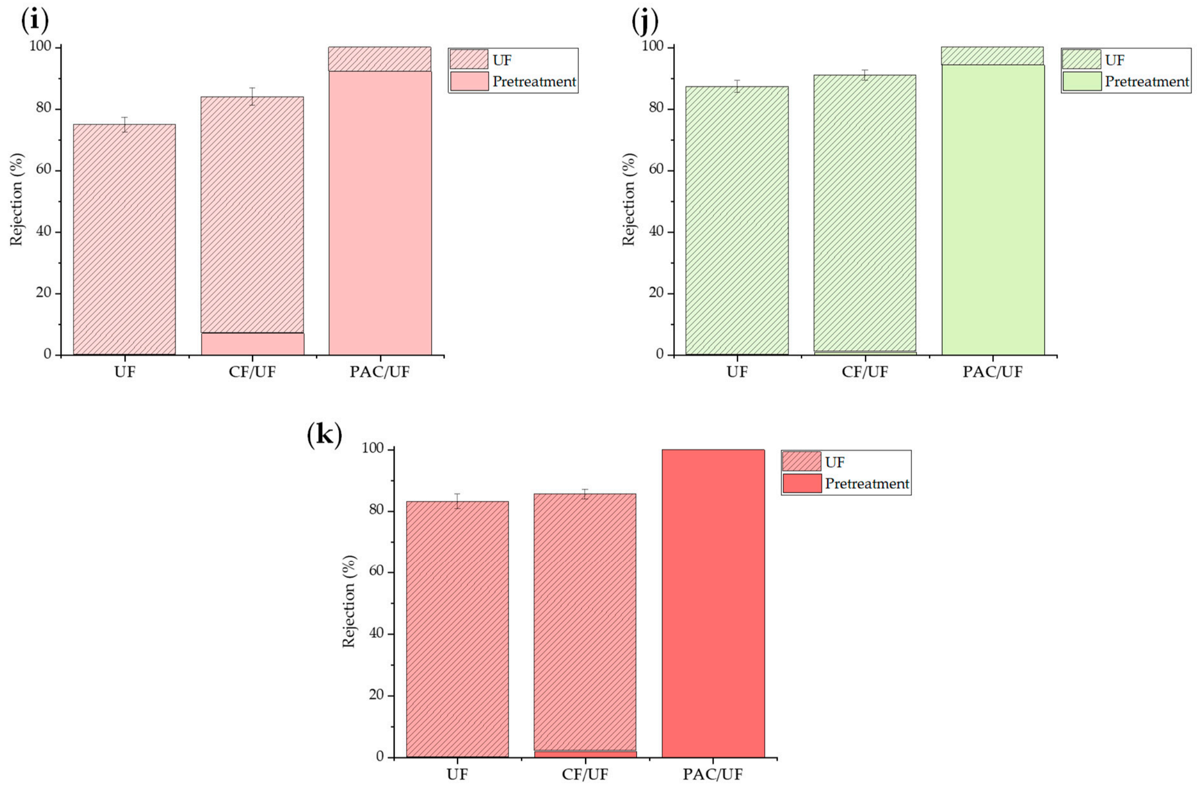

Figure S4 (cont.). Rejection of (**a**) AMX, (**b**) TMP, (**c**) DVF, (**d**) CFX, (**e**) FLZ, (**f**) SMX, (**g**) IMZ, (**h**) PCZ, (**i**) TBZ, (**j**) PNZ, (**k**) DTB in the UF of SE1 and total removal in the combined treatments CF/UF and PAC/UF.
